# Strangulated small bowel obstruction caused by isolated obturator nerve and pelvic vessels after pelvic lymphadenectomy in gynecologic surgery: two case reports

**DOI:** 10.1186/s40792-022-01459-w

**Published:** 2022-05-30

**Authors:** Riko Ideyama, Yoshihisa Okuchi, Kenji Kawada, Yoshiro Itatani, Rei Mizuno, Koya Hida, Kazutaka Obama

**Affiliations:** 1 Department of Surgery, Graduate School of Medicine, Kyoto University, 54 Shogo-in Kawaracho, Sakyo-ku, Kyoto, 606-8507 Japan; 2 Department of Gastroenterological Surgery and Oncology, Kitano Hospital Medical Research Institute, 2-4-20 Ogimachi, Kita-ku, Osaka, 530-8480 Japan; 3 Department of Surgery, Uji-Tokushukai Medical Center, 145 Makishima-cho Ishibashi, Uji, Kyoto 611-0042 Japan

**Keywords:** Pelvic lymphadenectomy, Strangulated small bowel obstruction, Obturator nerve, Minimally invasive surgery

## Abstract

**Background:**

Although small bowel obstruction (SBO) is a major complication occurring after abdominal surgery, few reports have described strangulated SBO after pelvic lymphadenectomy (PL). This report describes two cases of strangulated SBO caused by a skeletonized obturator nerve and pelvic vessels after laparoscopic PL during gynecologic surgery.

**Case presentation:**

Case 1: A 57-year-old woman with endometrial cancer underwent a laparoscopic semi-radical total hysterectomy with PL. Nine months after the operation, she visited our emergency room complaining about subacute pain spreading in the right groin, right buttock, and dorsal part of the right thigh. She had no abdominal pain. Although her symptoms were not typical, computed tomography (CT) revealed strangulated SBO in the right pelvis. Laparoscopic surgery revealed that the small bowel was ischemic. Then we converted to open surgery. We transected the right obturator nerve and umbilical artery, which constructed an internal hernia orifice in the right pelvis, followed by resection of the ischemic small bowel. Fortunately, during 6-month follow-up, she showed only slight difficulty in walking as a postoperative complication. Case 2: A 62-year-old woman with cervical cancer underwent laparoscopic radical hysterectomy with PL. Six months after the operation, she visited our hospital emergently because of sudden onset of abdominal pain and vomiting. CT showed strangulated SBO. Urgent laparoscopic surgery exhibited the incarcerated small bowel at the right pelvis. Consequently, we converted to open surgery. The terminal ileum was detained into the space constructed by the right umbilical artery. We cut the umbilical artery and performed ileocecal resection. After the surgery, she was discharged with no complication or sequela.

**Conclusion:**

When examining a patient after PL who complains of severe pain or symptoms, one should consider the possibility of PL-related SBO, even if the pain is apparently atypical for SBO.

## Background

Pelvic lymphadenectomy (PL) is intended as a complete cure of multiple malignant diseases in the pelvic cavity [1–6]. Recently, minimally invasive surgery using laparoscopic and robot-assisted modalities have also been applied to this surgical procedure [7–10]. Strangulated small bowel obstruction (SBO) induced by exposed vessels and nerves in the pelvic cavity is a rare complication after PL. Rapid diagnosis is crucially important. Nevertheless, diagnosing them accurately is much more challenging if they present atypical symptoms. This report describes two rare cases of PL-related strangulated SBO after laparoscopic PL. One patient showed typical symptoms and radiological findings as incarcerated SBO, whereas the other displayed atypical symptoms such as severe pain in the dorsal part of the thigh with no abdominal pain, which was difficult to diagnose accurately as strangulated SBO.

## Case presentation

### Case 1

**Fig. 1:**
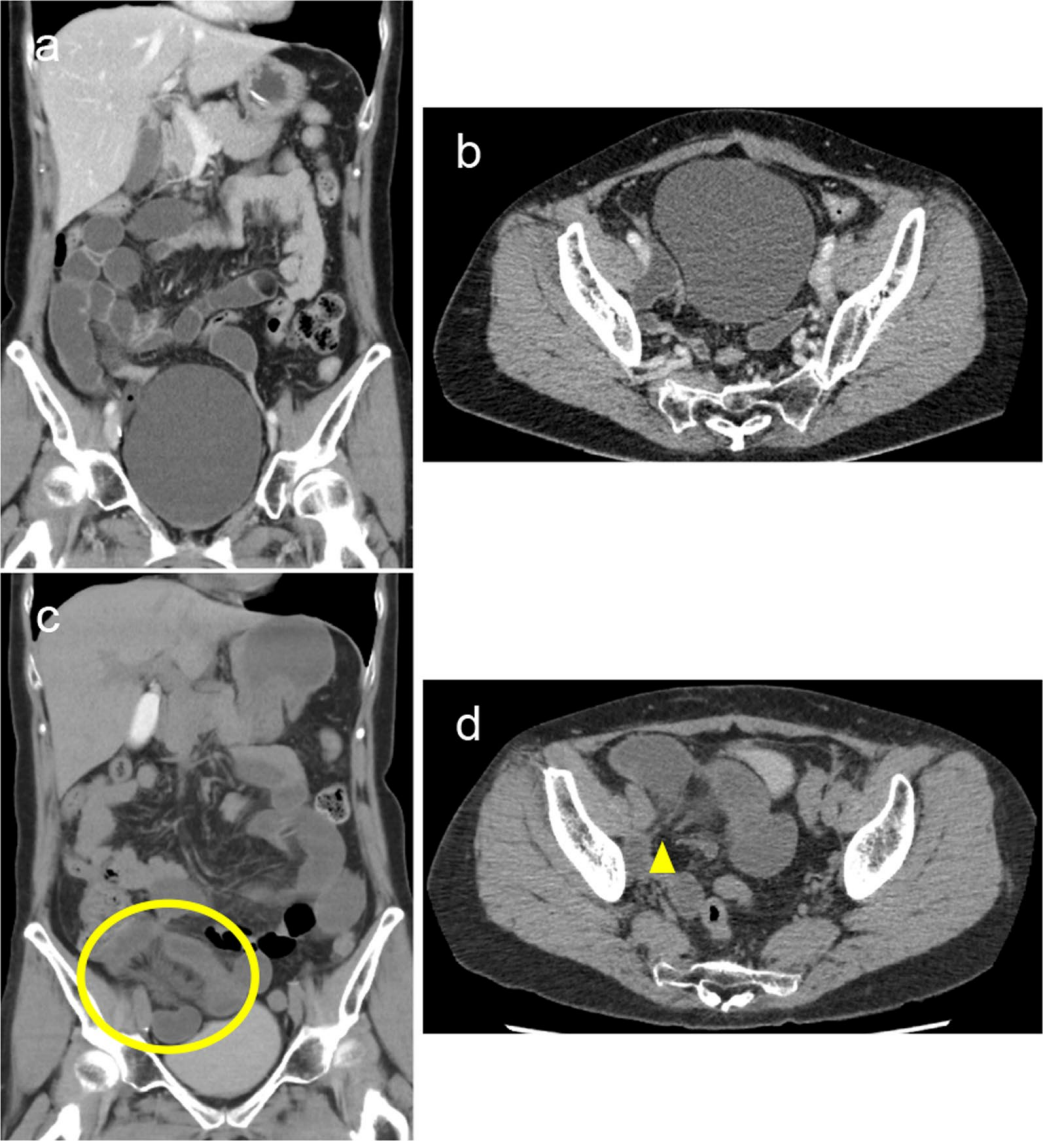
Abdominal CT scan images of the case 1 patient taken at her first visit (**a**, **b**) and 6 h after hospitalization (**c**, **d**). **a** and **b** Coronal (**a**) and axial (**b**) enhanced CT scan images at her first visit showed dilated small intestine without a closed loop. **c** and **d** Coronal (**c**) and axial (**d**) plain CT scan images 6 h after the hospitalization exhibited edematous mesentery (yellow circle) and a closed loop, the origin of which was in the right pelvic wall (yellow arrowhead)

A 57-year-old woman (body mass index, 18.9 kg/m^2^) visited our emergency room for the chief complaint of subacute pain spreading in the right groin, right buttock, and dorsal part of the right thigh. Regarding the abdominal findings, she showed no tenderness or distention. Nine months prior, she had undergone a laparoscopic semi-radical total hysterectomy with bilateral salpingo-oophorectomy, pelvic lymphadenectomy, para-aortic lymphadenectomy, partial omentectomy, and peritoneal stripping for endometrial cancer. After surgery, she received standard systemic adjuvant chemotherapy for 6 months. No recurrent sign was observed. When she visited our hospital, we first suspected sciatica from her chief complaint. Computed tomography (CT) images of the abdomen and pelvis were obtained to ascertain the causes of her chief complaint. Unexpectedly, CT images revealed that small bowel obstruction (SBO) without a closed-loop had occurred in the right pelvic wall (Fig. 1a and b). At this time point, however, her pain had completely disappeared because of the administration of an analgesic. She therefore reported no abdominal pain. The laboratory data indicated moderate inflammation, WBC 10,110/μL, and lactate level 28.8 mg/dL. These clinical data and symptoms were atypical and insufficient to diagnose her as having strangulated SBO. Therefore, we chose hospitalization to facilitate close follow-up of her general condition. Six hours after hospitalization, she became affected again by acute, similar severe pain in the dorsal part of the right thigh. Follow-up CT presented more edematous mesentery of the small bowel and an increase of ascites (Fig. 1c and d), strongly indicating that the strangulated SBO was worsening.

**Fig. 2:**
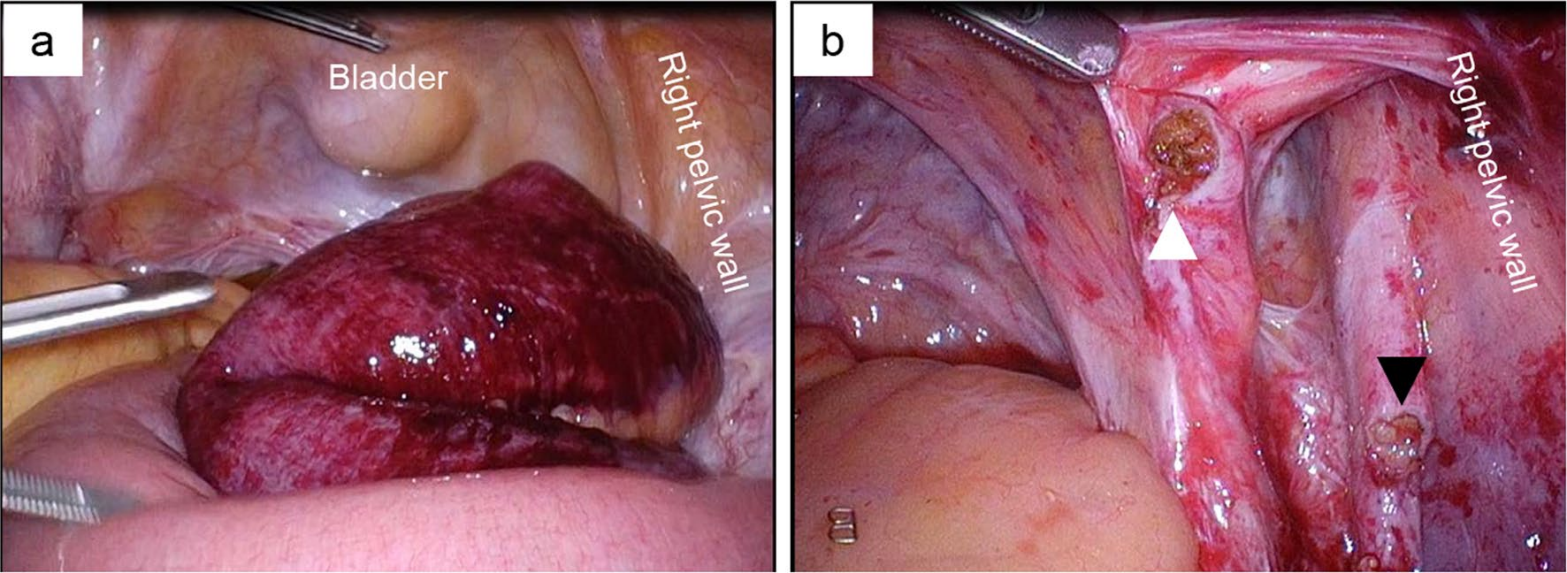
Laparoscopic views in the pelvic cavity in case 1 patient. **a** Strangulated small bowel displayed dark red color, indicating severe ischemia. **b** Umbilical artery stump (white arrowhead) and obturator nerve stump (black arrowhead) after resection of incarcerated small bowel and bands

Therefore, we performed emergent laparoscopic surgery and found the strangulated small bowel with bloody ascites in the right pelvis. The strangulated small bowel appeared to be severely ischemic (Fig. 2a). The dilated oral side of the small bowel prevented us from grasping it with laparoscopic forceps safely and from making a clear surgical view. Therefore, we converted to open surgery to confirm the cause of the strangulated ileus. Results showed two isolated bands in the right pelvic region, which caused the internal hernia orifice of the strangulated small bowel. We were unable to help transecting both to release the strangulated small bowel. The incarcerated small bowel was finally resected because the blood flow did not recover. After resection of the incarcerated small bowel, it became clear that the bands constructing the internal hernia orifice were the right obturator nerve and umbilical artery (Fig. 2b). Although the initial gynecologic surgery had been highly invasive, none of the small bowel adhered to itself or to the abdominal walls. Adhesion-preventing material had been used before completing that operation. The patient progressed favorably after the operation. She was discharged with no major complication on the 6th postoperative day. Presently, she has right adductor muscle weakness (manual muscle test: MMT 4/5), but showed only slight walking difficulties at 6-month follow-up.

### Case 2

**Fig. 3:**
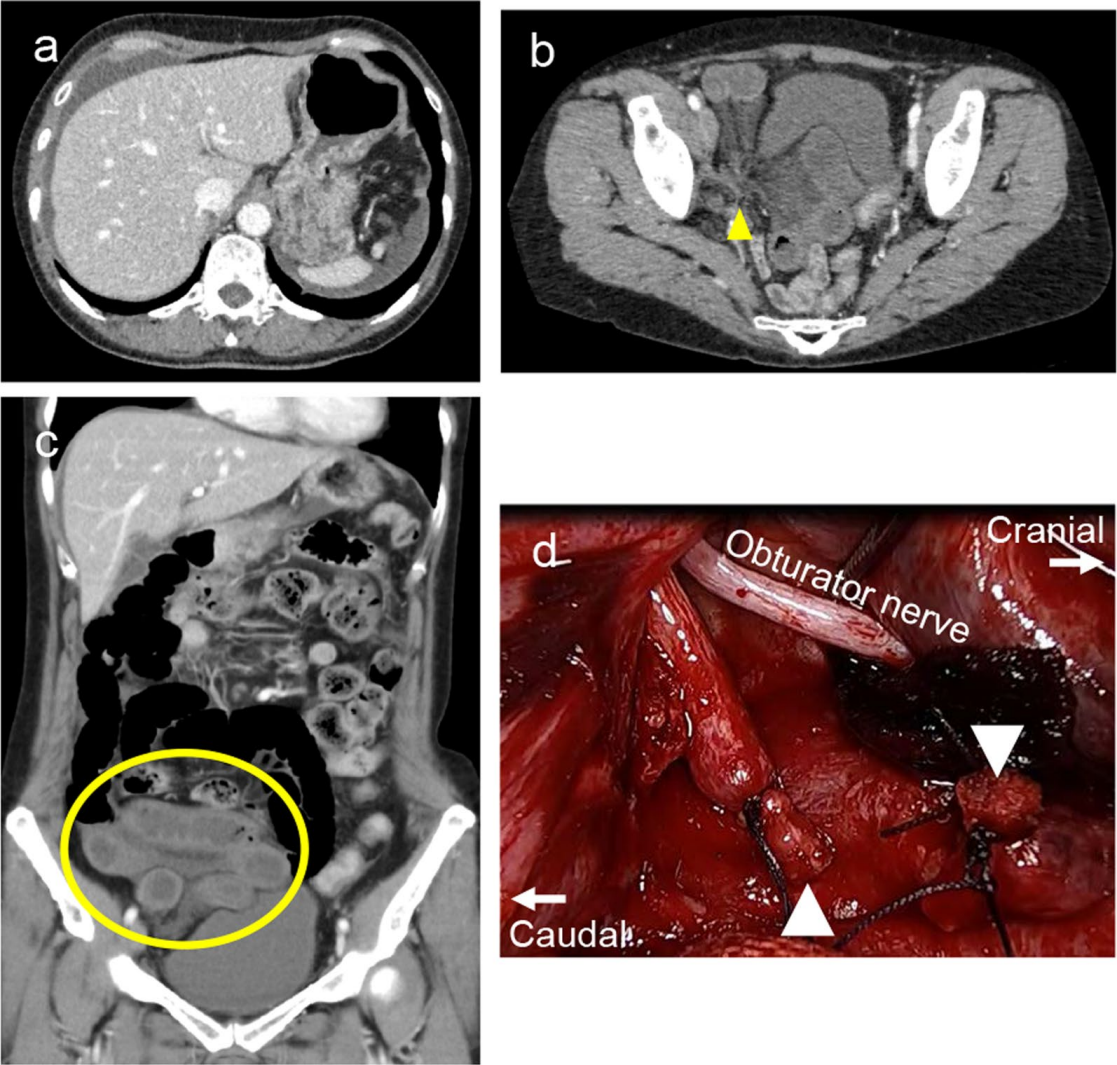
Abdominal CT scan images before urgent surgery (**a**–**c**) and a laparoscopic view after resection of umbilical artery (**d**) of the case 2 patient. **a** Massive ascites are found on the liver surface. **b** and **c** Axial (**b**) and coronal view (**c**) of the strangulated small bowel. The yellow arrowhead and circle denote the strangulated origin and edematous mesentery and intestine. **d** Umbilical artery stump (white arrowhead) after resection of the incarcerated small bowel and a cord

A 62-year-old woman (body mass index, 20.6 kg/m^2^) visited our hospital emergently because of sudden onset of abdominal pain and vomiting. Six months prior, the patient had undergone laparoscopic radical hysterectomy with bilateral salpingo-oophorectomy and pelvic lymphadenectomy for cervical cancer and showed no recurrence signs. Laboratory data exhibited marked elevation of the white blood cell count (15,760/μL) and the lactate level (49.5 mg/dL). Enhanced abdominal CT scan demonstrated massive ascites, edematous mesentery, and ischemic small bowel near the right pelvic wall, strongly suggesting the possibility of strangulated SBO (Fig. 3a–c). Immediately after starting urgent laparoscopic surgery, massive bloody ascites and incarcerated small bowel were observed (Fig. 3d). As in case 1, the small intestine did not adhere to itself or to the abdominal wall. Adhesion-preventing material had also been used in gynecologic surgery. The incarcerated small bowel was severely dilated and was apparently ischemic ultimately. Therefore, after converting to open surgery, we found the terminal ileum detained into the space constructed by the right umbilical artery. The right umbilical artery was resected to extract the ischemic small bowel. Ileocecal resection was performed. She was discharged with no important complication on the 7th postoperative day.

## Discussion

We experienced two cases of strangulated SBO after PL during gynecologic surgery. In both cases, emergent operations were necessary, with cutting of the bands constructing the internal hernia orifice and resecting the incarcerated small bowel. The bands in the first case were the right umbilical artery and obturator nerve, whereas the other was the right umbilical artery. Ascertaining the cause of the strangulated SBO clearly before the operations was challenging. Especially in the first case, the patient displayed atypical symptoms such as severe pain in her right buttock with no abdominal pain. Retrospectively, we inferred the pain she exhibited as similar to Howship–Romberg sign in a patient with an obturator hernia [11].

PL is a standard surgical procedure performed for several malignant diseases affecting the pelvic organs, such as ovarian [1], cervical, endometrial [2, 3], prostate [4], bladder [5], and rectal cancers [6]. Strangulated internal hernia involving the right common iliac artery after PL in a patient with testicular cancer was first reported in 1978 [12]. No report for 30 years thereafter described strangulated internal hernia related to skeletonized vessels or nerves after PL. In 2008, Kim et al. reported strangulated internal hernia involving the right external iliac artery in a patient with cervical cancer [13]. To date, a total of 19 cases have been described in 17 reports, including ours (Table 1). We assume that the recent increase of case reports related to PL-related SBO might be attributable to the development of adhesion-preventing materials [14–16] and to the wider utilization of minimally invasive surgery such as laparoscopic and robot-assisted surgery [7–10].

**Table 1:** Reported cases of strangulated small bowel obstruction after pelvic lymphadenectomy

Year	Age/sex	Cancer	Original approach	Duration	Hernia orifice	Treatment	Bowel resection
1978 [12]	52/M	Testicular	Open	4 months	Rt CIA	Open	Yes
2008 [13]	67/F	Cervical	Laparoscopic	3 months	Rt EIA	Open	Yes
2013 [17]	56/F	Ovarian	Laparoscopic	4 years	Lt EIA	Lap	No
2014 [18]	39/F	Cervical	Laparoscopic	2 years	Rt CIA	Lap to open	Yes
2015 [24]	50/M	Bladder	Robot	5 months	Rt CIA	Open	Yes
2016 [25]	50/M	Prostate	Robot	1 year	Lt EIA	Open	Yes
2018 [19]	38/F	Cervical	Laparoscopic	6 months	between Rt UA and ON	Lap	No
2018 [29]	68/M	Rectal	Laparoscopic	4 months	Rt SVA	N/A	Yes
2018 [29]	59/M	Rectal	Laparoscopic	2 months	Rt SVA	Lap	Yes
2018 [26]	64/M	Prostate	Robot	1 year	Rt EIA	Lap to open	Yes
2019 [27]	72/M	Prostate	Robot	2 months	Lt EIA	Open	Yes
2020 [30]	63/M	Rectal	Robot	1 month	Rt ON	Lap	Yes
2020 [28]	78/M	Bladder	Laparoscopic	38 months	Rt ON	Open	Yes
2020 [20]	68/F	Endometrial	Laparoscopic	7 years	between Rt EIA and EIV	Open	Yes
2020 [21]	53/F	Cervical	Laparoscopic	1 month	Rt SVA	Open	Yes
2021 [22]	46/F	Cervical	Laparoscopic	9 years	Lt EIA/V	Lap to open	No
2021 [23]	67/F	Ovarian	N/A	6 years	Rt EIA/V	Lap	No
2022	57/F	Endometrial	Laparoscopic	9 months	Rt UA/ON	Lap to open	Yes
2022	62/F	Cervical	Laparoscopic	6 months	Rt UA	Lap to open	Yes

Duration stands for the time from the original surgery to the internal hernia surgery

Lap, laparoscopic surgery; Open, open surgery; Robot, robot-assisted surgery; Rt, right; Lt, left; CIA, common iliac artery; EIA/V, external iliac artery/vein; UA, umbilical artery; ON, obturator nerve; SVA, superior vesical artery; N/A, not applicable

It is particularly interesting that about half of reports have originated from the gynecologic field, in which all the initial gynecologic surgeries were done laparoscopically [13, 17–23]; moreover, approximately one-third of those reports were from the urologic area, in which the primary operations, except for the first case reported, were laparoscopic or robot-assisted surgeries [12, 24–28]. Recently, two reports described three cases after rectal cancer surgeries [29, 30]. In general, PL performed in gynecologic and urological fields can dissect lymph nodes around an external iliac artery or common iliac artery [1, 2, 4]. However, these lymphadenectomies are not always done in rectal cancer [6]. Perhaps for that reason, among others, few related reports from the rectal cancer field have been published.

According to past papers, vessels or nerves constructing the internal hernia orifice were right common iliac artery (3 cases) [12, 18, 24], left external iliac artery and/or vein (4 cases) [17, 22, 25, 27], right external iliac artery and/or vein (4 cases) [13, 20, 23, 26], right superior vesical artery (3 cases) [21, 29] and right umbilical artery and/or obturator nerve (5 cases) [19, 28, 30]. In fact, PL-related SBO is more common on the right side than on the left side (Table 1), which might be attributable to the fact that the left side is covered with the sigmoid colon.

The median time to onset from PL was 6 months, but its distribution was from 2 to 108 months, underscoring the point that PL-related SBO can occur anytime in patients with a history of PL. Three of four cases in which the incarcerated small bowel was preserved were of laparoscopic techniques [17, 19, 22, 23], whereas open surgeries were performed in 13 of 15 cases in which the incarcerated small bowel was removed, including five cases converted from laparoscopic surgeries. Those findings might reflect the difficulty, in many cases, of providing the patients with an accurate diagnosis rapidly.

The patient in case 1 presented symptoms similar to the Howship–Romberg sign. Generally, there are relationships between the symptoms of obturator hernia and the thin body; however, previous reports did not refer to any connections between PL-related SBO and the lean body.

Other than our cases, only three published reports describe PL-related SBO caused by a band constructed by the obturator nerve [19, 28, 30]. In these cases, the authors preserved the obturator nerve, leaving the hernia orifice unrepaired. By contrast, we resected the obturator nerve in our case. Generally, damage to the obturator nerve can induce leg weakness and gait disorders. Some patients also present sensory symptoms or severe pain in the groin, buttock, and medial thigh [31, 32]. Ningshu et al. described that severe damage to the obturator nerve causes permanent neurological deficits and motor weakness [33]. At the same time, they described the possibility of using analgesics, physiotherapy, and obturator nerve blockade for obturator neuropathy. In fact, the patient in case 1 complained of right adductor muscle weakness (MMT 4/5) immediately after surgery, but it had almost disappeared at 6-month follow-up. Therefore, even if resection of the obturator nerve is unavoidable, conservative management should be considered to recover or alleviate symptoms after the operation.

## Conclusions

When examining a patient after PL who complains of severe pain or symptoms, we should consider the possibility of PL-related SBO, even if the pain is apparently atypical for SBO.

## Data Availability

Datasets supporting the conclusions of this article are included within the article.

## Abbreviations

SBO: Small bowel obstruction
PL: Pelvic lymphadenectomy
CT: Computed tomography
MMT: Manual muscle test
